# *CScape-somatic*: distinguishing driver and passenger point mutations in the cancer genome

**DOI:** 10.1093/bioinformatics/btaa242

**Published:** 2020-04-13

**Authors:** Mark F Rogers, Tom R Gaunt, Colin Campbell

**Affiliations:** 1 Intelligent Systems Laboratory, University of Bristol, Bristol BS8 1UB, UK; 2 MRC Integrative Epidemiology Unit (IEU), University of Bristol, Bristol BS8 2BN, UK

## Abstract

**Motivation:**

Next-generation sequencing technologies have accelerated the discovery of single nucleotide variants in the human genome, stimulating the development of predictors for classifying which of these variants are likely functional in disease, and which neutral. Recently, we proposed *CScape*, a method for discriminating between cancer driver mutations and presumed benign variants. For the neutral class, this method relied on benign germline variants found in the 1000 Genomes Project database. Discrimination could, therefore, be influenced by the distinction of germline versus somatic, rather than neutral versus disease driver. This motivates this article in which we consider predictive discrimination between recurrent and rare somatic single point mutations based solely on using cancer data, and the distinction between these two somatic classes and germline single point mutations.

**Results:**

For somatic point mutations in coding and non-coding regions of the genome, we propose *CScape-somatic*, an integrative classifier for predictively discriminating between recurrent and rare variants in the human cancer genome. In this study, we use *purely cancer genome data* and investigate the distinction between minimal occurrence and significantly recurrent somatic single point mutations in the human cancer genome. We show that this type of predictive distinction can give novel insight, and may deliver more meaningful prediction in both coding and non-coding regions of the cancer genome. Tested on somatic mutations, *CScape-somatic* outperforms alternative methods, reaching 74% balanced accuracy in coding regions and 69% in non-coding regions, whereas even higher accuracy may be achieved using thresholds to isolate high-confidence predictions.

**Availability and implementation:**

Predictions and software are available at http://CScape-somatic.biocompute.org.uk/.

**Contact:**

mark.f.rogers.phd@gmail.com or C.Campbell@bristol.ac.uk

**Supplementary information:**

Supplementary data are available at *Bioinformatics* online.

## 1 Introduction

Next-generation sequencing technologies have accelerated the discovery of single-nucleotide variants (SNVs) in the human genome, stimulating the development of predictors for classifying which of these variants are likely functional in disease, and which neutral. Predictors have been developed for variants in both coding and noncoding regions of the human genome. For example, in Shihab *et al.* (2015), we developed such a predictor based on pathogenic disease-driver germline variants from the Human Gene Mutation Database (*HGMD*) (Stenson *et al.*, 2014), and assumed neutral variants from the 1000 Genomes Project Consortium (1000G) (The 1000 Genomes Project Consortium, 2012). Multiple types of data may be informative, so we used an integrative binary classifier which weighted component data types according to their relative informativeness (Shihab *et al.*, 2015). A variety of similar predictors have been proposed (Adzhubei *et al.*, 2010; Kircher *et al.*, 2014; Kumar *et al.*, 2009; Liu *et al.*, 2017; Quang *et al.*, 2015; Reva *et al.*, 2011). In Rogers *et al.* (2017a), we proposed *CScape*, a classifier for predicting the driver status of SNVs in the human cancer genome with a follow-on investigation of biological insights in Darbyshire *et al.* (2019). By a *driver*, we mean a disease enabler, therefore including the sub-instances of gain-of-function, loss-of-function or both simultaneously.

As tumors evolve, they accrue thousands of somatic mutations that are commonly labeled according to their role in cancer development: *driver mutations* are subject to positive selection during a tumor’s evolutionary progress, as they confer a growth advantage and contribute to tumor growth. *Passenger mutations* accumulate as tumors evolve, and may confer no advantage or may even inhibit tumor fitness (Pon and Marra, 2015; Stratton *et al.*, 2009). Oncogenesis is believed to be caused by a small number of key driver mutations (Darbyshire *et al.*, 2019; Martincorena *et al.*, 2017) that trigger tumor growth and induce subsequent passenger mutations as tumors proliferate (Bozic *et al.*, 2010; McFarland *et al.*, 2014; Pon and Marra, 2015). Many more passenger than driver mutations exist in cancer cells and distinguishing between the two classes remains a significant challenge (Marx, 2014). Germline mutations have been identified as drivers in genes such as *BRCA1* and *BRCA2*, but it is estimated that up to 90% of cancer-related genes are influenced by somatic mutations: those that accrue during a patient’s lifespan (Futreal *et al.*, 2004). Furthermore, the immune system could be expected to tolerate germline mutations but remove cells with particular types of somatic mutation, leading to differing distributions between germline and somatic variation. Hence, understanding particular characteristics that differentiate somatic and germline mutation will be crucial to our understanding of how the disease progresses.

In this article, we focus on a machine learning approach to distinguishing between driver and passenger SNVs across the human cancer genome. The development of such classifiers will be important for interpreting cancer sequence databases currently being compiled, such as the Cancer Genome Atlas (Weinstein *et al.*, 2013), the International Cancer Genome Consortium (ICGC) (Zhang *et al.*, 2011) and national programmes, such as the Genomics England (100 000 genomes) Project. Mirroring previous methods (Rogers *et al.*, 2015, 2017a; Shihab *et al.*, 2015), we use an integrative classifier and select features from a wide variety of data sources. Using leave-one-chromosome-out cross-validation (LOCO-CV), the proposed method, which we call *CScape-somatic*, outperforms alternative models, achieving balanced test accuracies of 74% in coding regions and 69% in non-coding regions.

We also associate a confidence measure to the predicted class assignments (cf. Supplementary Section S1). To interpret this confidence measure, in Supplementary Section S4, we consider two thresholds, a default threshold and a high-confidence threshold. If we restrict prediction to highest confidence instances only (*cautious classification*) then balanced accuracy in LOCO-CV increases to 92% for coding regions and 87% for non-coding regions, though with this level of test accuracy is confined to 10% of coding and 9% of non-coding nucleotide positions across the genome, respectively.

## 2 Materials and methods

### 2.1 Recurrence thresholds

**Fig. 1.**
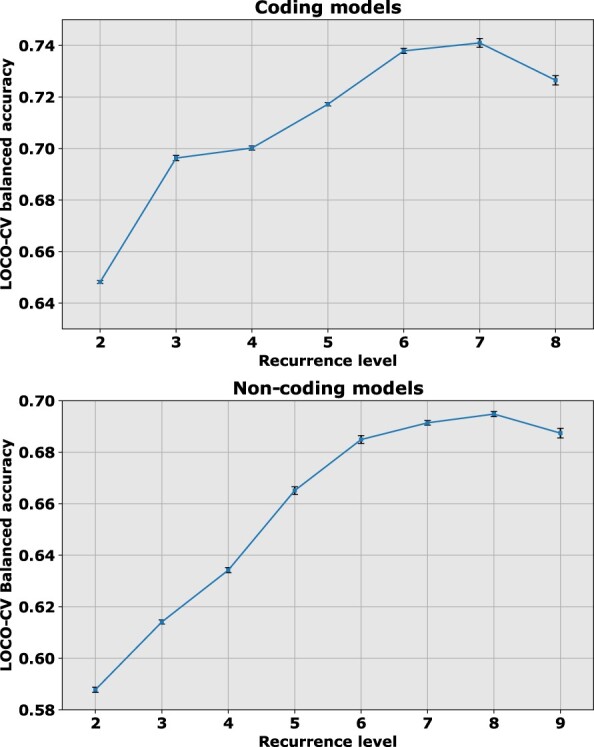
Balanced accuracy for models at different COSMIC recurrence levels shows that the coding models achieve a peak validation accuracy of 74.1% at a recurrence threshold of *ρ* = 7 (top), whereas the non-coding models achieve a peak accuracy of 69.5% at a recurrence threshold of *ρ* = 8 (bottom)

We assembled two datasets based on variants found in the COSMIC database (version 84, February 2018) (Forbes *et al.*, 2010). Among the COSMIC database annotations is the recurrence level, or the number of times a mutation has been observed in different cases. In the discussion below, *highly recurrent* variants have a recurrence of r≥ρ, where we select *ρ* = 8 in non-coding regions and *ρ* = 7 in coding regions. The dependence of predictive accuracy on unseen validation data, versus recurrence level *r*, is depicted in Figure 1. For somatic variants, the other category of interest will be *rare somatic* SNVs which occur once in the whole dataset (*r* = 1). These two categories of somatic alterations will contain variants with differing disease-driver statuses. It is reasonable to assume that some highly recurrent variants, specific to cancer samples and absent from healthy individuals, are actually neutral passengers. A recurrent somatic SNV could be closely co-located within a region where there is an active disease driver. Similarly, a rare somatic SNV (*r* = 1) could actually be a rare driver. However, it is plausible to assume that *recurrently observed somatic* SNVs, which are restricted to cancer samples, are enriched for driver mutations. Similarly, *rare somatic* SNVs could be expected to be enriched for neutral variants. Even if this statement were challenged, we point out that the consequence of this study is to show that membership of these two classes can be predicted with a non-trivial accuracy on unseen test data, and hence these two classes must have different enrichments and characteristics. Our interest in discriminating *recurrent somatic* SNVs from *rare somatic* SNVs is therefore that it provides an alternative insight beyond a discrimination between germline neutrals (from healthy individuals) and recurrent somatic variants from cancer patients, absent from healthy individuals (Rogers *et al.*, 2017a). This latter distinction could be influenced by a bias toward germline versus somatic discrimination, rather than the intended distinction of passenger versus disease driver.

A further possible bias may be introduced if our class of negative examples, say the rare variants, are located in different genomic regions from the positive examples, the highly recurrent variants. For example, the positives may appear predominantly near transcription start sites while negatives are distributed more broadly (Kircher *et al.*, 2014; Ritchie *et al.*, 2014; Shihab *et al.*, 2015). To ensure the locations of rare somatic mutations approximate those of recurrent somatic mutations, we select only those rare mutations found within a window *w* of a recurrent mutation. For coding examples, we use *w *=* *10 000, and for non-coding examples, we use *w *=* *1000 (Supplementary Section S1). Hence our final training sets, outlined in Supplementary Tables S1 and S2, consist of 27 575 coding examples and 10 908 non-coding examples.

### 2.2 Feature groups

All of our data are based on the GRCh37/hg19 version of the human genome and detailed further in Supplementary Material. Following our previous work (Rogers *et al.*, 2015, 2017a,b; Shihab *et al.*, 2015), we annotated our datasets using more than 30 *feature groups* that could be predictive of pathogenicity. For discriminating between somatic variants, we found the following feature categories to be predictive:

- *Genomic*: genomic features include GC content, local mutation frequency (Martincorena and Campbell, 2015), sequence spectra (Leslie *et al.*, 2002), proximity to gene features, such as splice sites or transcription factor binding sites, predicted functional elements and measures of region uniqueness.
- *Evolutionary*: evolutionary features include a comprehensive set of conservation-based measures provided by tools, such as PhastCons (Siepel *et al.*, 2005), PhyloP (Pollard *et al.*, 2010) and FATHMM (Shihab *et al.*, 2015).
- *Consequences* (*coding only*): using the Variant Effect Predictor (McLaren *et al.*, 2016), we use binary vectors to represent allele consequences and the affected amino acids within all transcripts associated with a mutation.

The COSMIC database also provides a set of *mutational signatures* that are specific to oncogenic mutations. These are associated with various distinct forms of mutation, such as DNA replication errors, defective DNA repair, enzymatic DNA modification and exposure to mutagens (Alexandrov *et al.*, 2013). However, this signature set is still evolving and may represent only a subset of potential oncogenic driver signals. Furthermore, metrics used to derive some of these signatures are based in part on drivers gleaned from the COSMIC database and potentially could bias our models. Hence, our final models use seven distinct feature groups: *Conservation*, *GC content*, *Sequence uniqueness*, *Local mutation frequency*, *Proximity to gene features*, *Spectrum* and *Functional elements*. More detailed descriptions of these feature groups, and the machine learning method used, appear in Supplementary Material and in Rogers *et al.* (2017a).

### 2.3 *CScape-somatic* models

We evaluated all models using LOCO-CV testing (Table 1), omitting mitochondrial and allosomal (X and Y) chromosomes from testing as these have evolutionary characteristics distinct from autosomal chromosomes, and tend to yield fewer examples. For each fold, we leave out one test chromosome while the remaining 21 chromosomes are used to train the model, using the same model parameters for all folds. Except where noted, we trained models using randomly selected, balanced sets of 4000 positive and 4000 negative examples. This smaller subset of examples yields accuracy nearly as high as with complete training sets but takes less time to train, and allows us to estimate the variability of test results across multiple LOCO-CV runs. For testing, we used all available examples for the left-out chromosome, resulting in slightly unbalanced test sets for coding and non-coding (Supplementary Table S2). For the training datasets, we balanced examples by class, and report results for balanced training for all test set estimations.

**Table 1.** Statistics for *CSS-noncoding* and *CSS-coding* applied to LOCO-CV test data provide estimates of how the models are likely to perform on new examples

Classifier	Bal. Acc.	Sens.	Spec.	MCC	PPV
*CSS-noncoding*	0.69	0.64	0.74	0.38	0.73
Cautious (τ=0.84)	0.84	0.87	0.81	0.67	0.91
*CSS-coding*	0.74	0.72	0.77	0.48	0.76
Cautious (τ=0.91)	0.92	0.96	0.88	0.85	0.93

*Note*: Shown are the performance statistics for each model: sensitivity (**Sens.**, the proportion of positive examples correctly classified), specificity (**Spec.**, the proportion of negative examples correctly classified), balanced accuracy (**Bal. Acc.**), the Matthews correlation coefficient (**MCC**) and the positive predictive value (**PPV**, the proportion of positive predictions that are true positives). *τ* is the cutoff on the confidence for cautious classification.

We integrated data from the feature groups outlined above and used them to train two distinct sub-classifiers: one for coding regions (*CSS-coding*), and a second for non-coding regions (*CSS-noncoding*). The simplest kernel method for integrating different data sources is to combine features from all sources into a single kernel. In previous work (Rogers *et al.*, 2017a,b), we have found that this approach yields excellent performance that may surpass multiple kernel methods (Rogers *et al.*, 2017b), as single kernel methods allow models to learn interactions between features from different sources. Given at least 30 possible data sources, the number of possible combinations of feature groups makes exhaustive testing impractical. Instead, we use a forward selection approach based on previous work in which we found that sequential learning could be an effective means to identify an optimal combination of feature groups (Rogers *et al.*, 2015). To identify the data sources to include in each model, we first rank all feature groups by balanced accuracy. Starting with the top-ranked feature group by validation accuracy, we iterate over the remaining feature groups, creating models by combining each of the remaining groups with the top-ranked group to form a single kernel. If any of these models yield higher balanced accuracy than the best model, it becomes the new best model. We continue this process until none of the subsequent models yields significantly higher balanced accuracy than the current best model in LOCO-CV (Supplementary Fig. S1). We evaluate all combinations with and without data normalization, where we standardize features by subtracting the mean and dividing by the standard deviation. For these models, we observed no difference in performance between the raw feature values and standardized data. The final *CSS-noncoding* model includes five feature groups: *Conservation*, *Local mutation frequency*, *Distance from gene features* and two related to sequence: *GC content* and *Sequence uniqueness*. For *CSS-coding*, the best model uses all of the feature groups used in *CSS-noncoding* plus the *Functional elements* and *Spectrum* groups (Supplementary Section S2).

## 3 Results

### 3.1 Measurable differences between germline and somatic neutral variants

The methodology we use will be similar to that used with *CScape* (Rogers *et al.*, 2017a). However, the key difference is that we wish to explore the potential for discriminating between two different classes of somatic variants: highly recurrent SNVs, which we label as *positives*, and rare SNVs which we label as *negatives*. The other distinction is between the neutral germline variants we used to train our *CScape* models and the *r *=* *1 somatic SNVs in cancer samples. To investigate this latter distinction, we evaluated 30 different feature groups to detect differences between these latter two classes of variants.

### 3.2 Non-coding data: germline versus somatic

**Fig. 2.**
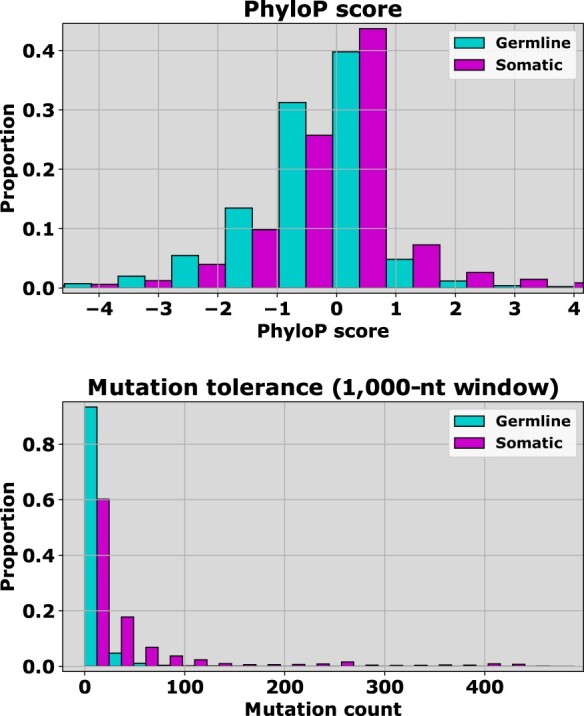
Scoring distributions for SNVs in the **non-coding** datasets show differences between *germline* (1000 Genomes) and *rare somatic* (COSMIC, *r *=* *1) examples. The features that discriminate most clearly between germline and somatic variants are those associated with conservation scores (top) and the somatic mutation frequency within a local region (bottom). Conservation scores do not yield the kind of discrimination we see typically when comparing pathogenic or oncogenic mutants with presumed benign variants, however PhyloP scores suggest that putative somatic passenger variants are more closely associated with highly conserved regions (lower scores indicate greater conservation) than benign germline variants (top). This same pattern holds for other conservation scores, but the distinction is less clear (Supplementary Fig. S2). Somatic variants also appear to reside in regions with higher mutation tolerance, as measured by the number of somatic variants found within a region of 1000 positions (bottom). The individual probabilities that the two distributions in each subplot come from the same underlying distribution are upper bounded by $10^{-18}$, and hence the differences are certainly statistically significant

In non-coding regions, several feature groups yielded different distributions for *r *=* *1 somatic variants and germline neutral variants. These are depicted in Figure 2 and Supplementary Figure S2, and the distinction is highly significant by hypothesis testing. For example, PhyloP conservation scores for *r *=* *1 somatic variants tend to be higher (associated with more highly conserved regions) and fall within a narrower range than neutral germline variants (Fig. 2, top). Based on our mutation tolerance measure, *r *=* *1 somatic variants reside in regions where somatic variants typically cluster, while benign germline variants appear in these regions less often (Fig. 2, bottom). These patterns are consistent with other features in the same groups (Supplementary Fig. S2), and hence supports our hypothesis that by developing models focused solely on somatic variants, we may begin to tease out differences between cancer drivers and putative passenger variants. However, one should be cautious about drawing inferences from these results. For example, germline neutral variants have higher percent GC content scores in coding regions, but lower scores in non-coding regions, so it is unclear whether GC content plays a significant role, or whether it merely correlates with other features.

### 3.3 Coding data: germline versus somatic

Conservation estimates feature prominently in many methods designed to predict pathogenic or oncogenic variants in coding regions of the genome, including our own FATHMM-MKL (Shihab *et al.*, 2015), FATHMM-XF (Rogers *et al.*, 2017b) and *CScape* (Rogers *et al.*, 2017a). The selection of positive examples (pathogenic or oncogenic) is relatively clear, but selecting appropriate neutral examples may be challenging. Hence, we used conservation scores to assess characteristic differences between neutral germline and somatic variants. For our analysis, we use three different methods for scoring conserved positions in a genome: *PhastCons* (Siepel *et al.*, 2005), *PHYLOP* (Pollard *et al.*, 2010) and *FATHMM* (Shihab *et al.*, 2013). *PhastCons* produces scores that correspond to the probability that a particular position is in a conserved region: high scores correspond to high conservation probability. *PHYLOP* yields scores in a broader range, but positive scores generally correspond to conserved regions and negative scores, to variable regions. *FATHMM* scores also span a relatively broad range. In this case, negative scores correspond to conserved regions and positive scores reflect variable regions.

**Fig. 3.**
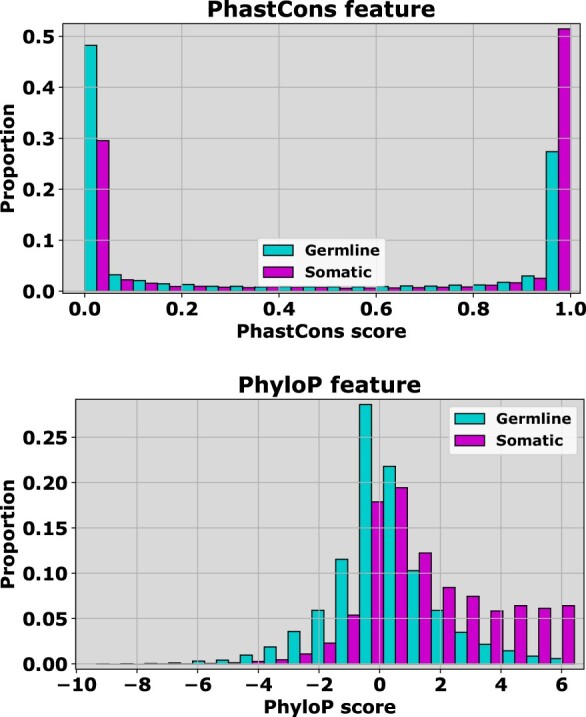
Two methods for estimating conservation in **coding** regions show that there are differences in scoring distributions between *germline* (1000 Genomes) and *rare somatic* (COSMIC, *r *=* *1) variants. With PhastCons scores (top) germline neutral variants tend to have low scores associated with more highly conserved regions, whereas somatic neutral variants tend to have higher scores. PHYLOP scores (bottom) exhibit a similar pattern where again, high scores are associated with conserved regions, whereas low scores are associated with more variable regions. While these differences are subtle, this suggests that developing a coding region classifier strictly based on somatic variants may yield better specificity for cancer drivers than the current *CScape* coding model. The individual probabilities that the two distributions in each subplot come from the same underlying distribution are upper bounded by $10^{-18}$, and hence the differences are certainly statistically significant

In coding regions, conservation scores tend to yield good discrimination between pathogenic and benign germline variants (Rogers *et al.*, 2017b; Shihab *et al.*, 2015), or between somatic driver and neutral germline variants (Rogers *et al.*, 2017a). Hence, it is not surprising that several conservation scoring methods also exhibit different distributions between rare somatic variants and neutral germline variants in coding regions (Fig. 3). Here, we show the results for two methods: *PhastCons* (Siepel *et al.*, 2005) and *PHYLOP* (Pollard *et al.*, 2010) [we find similar results for scores from *FATHMM* (Shihab *et al.*, 2013), Supplementary Fig. S3]. For conservation scores, we found that putative somatic passenger variants tend to have score distributions associated with more highly conserved regions than neutral germline variants. Note that, we observed the same pattern in conservation scores for non-coding variants, where rare somatic variants were also associated with more highly conserved regions (Fig. 2). These results are consistent with the idea that germline variants under selective pressure occur less frequently in conserved regions that are intolerant to variation. By contrast, rare somatic variants are under little or no selective pressure once tumors proliferate, and hence may tend to arise in conserved regions with a greater frequency.

### 3.4 Classifying recurrent and rare somatic variants

3.5 X indicate non-coding

### 3.5.1 Classifying somatic variants in non-coding regions

Cancer specific predictors have been proposed for prediction in coding regions of the cancer genome (Adzhubei *et al.*, 2010; Kumar *et al.*, 2009; Wong *et al.*, 2011). General purpose predictors have also been proposed for prediction across the entire genome (coding and non-coding regions) using catalogued disease drivers across a variety of disease traits [e.g. HGMD (Stenson *et al.*, 2014)], and recently, we have seen the emergence of classifiers designed to discriminate between cancer drivers and presumed benign variants from germline databases (Fu *et al.*, 2014; Rogers *et al.*, 2017a). However, there is currently a lack of predictors specifically trained to discriminate between somatically acquired putative drivers and passengers, particularly for non-coding regions of the cancer genome.

**Fig. 4.**
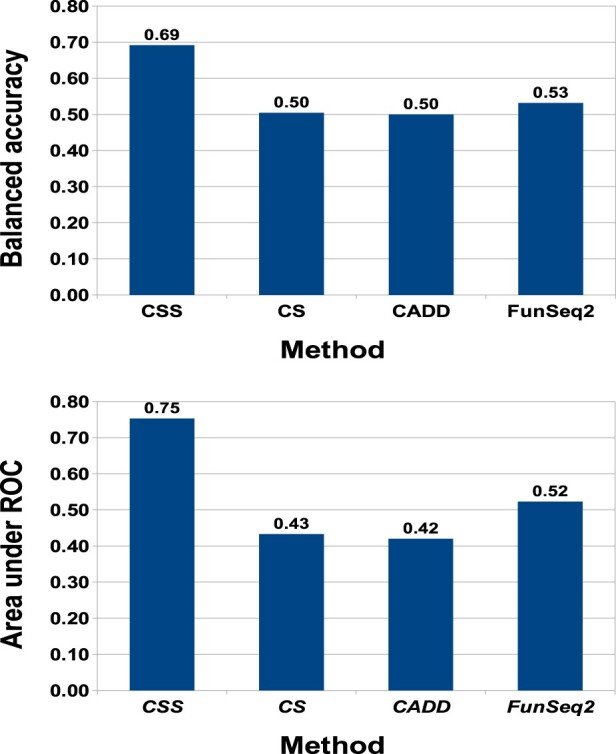
Comparison between *CScape-somatic* performance in LOCO-CV (**non-coding** regions, **COSMIC** data) with prediction results for *CScape*, *CADD* and *FunSeq2* on the same examples (CSS= *CScape-somatic* and CS= *CScape*). Top: *CScape-somatic* dramatically outperforms other methods on the COSMIC training data with accuracy over 69%. Of the other methods, only *FunSeq2* appears to yield prediction accuracy better than chance, at 52.7%. The remaining methods fare poorly, including the original *CScape*. Bottom: We see the same trend with ROC scores, as *CScape-somatic* yields satisfactory ranking performance of 0.75, whereas only *FunSeq2* yields rankings better than chance

Here, we consider the distinction between rare somatic variants and highly recurrent somatic variants, with the working assumption that the former class is enriched for neutral passengers while being distinct from germline neutrals, and with the latter class enriched for drivers. In Figure 4, we present results demonstrating that *CSS-noncoding* outperforms rival prediction tools for this distinction, based on the use of COSMIC data, both in terms of accuracy (top) and area-under-ROC-curve (AUC) score (bottom). In comparison with general-purpose classifiers such as CADD (Kircher *et al.*, 2014), and cancer-specific methods such as *CScape* (Rogers *et al.*, 2017a) and FunSeq2 (Fu *et al.*, 2014), our *CScape-somatic* model yields dramatically higher accuracy and AUC performance. *CScape-somatic* test accuracy with LOCO-CV is 69.2% while its nearest competitor, FunSeq2 yields 52.7%. Similarly, *CScape-somatic* yields an AUC score of 0.73 substantially higher than its nearest competitor, FunSeq2, with 0.52.

### 3.5.2 ICGC test data

ICGC data include patient identifiers, which enables us to find cancer variants that occur more than once. Hence, this dataset provides a good independent test for models that might discriminate between putative driver mutations (those found in multiple patients) and rare, prospectively neutral, mutations (those found just once). Within the ICGC data, we found 52 825 examples in non-coding regions after we applied our strict filtering criteria. This procedure yielded 37 802 variants associated with only one patient, and 15 023 examples associated with two or more patients. We selected positive examples using three different recurrence levels: r≥2, r≥3 and r≥4 (we found no examples associated with more than four patients). In each case, we restricted rare variants to be within 1000 nucleotide positions of highly recurrent putative driver, to mitigate potential bias related to genomic locations. This yielded 37 802 rare variants and 15 023 recurrent variants at r≥2, 3781 rare variants and 1548 recurrent variants at r≥3 and 1207 rare variants and 481 recurrent variants at r≥4.

**Fig. 5.**
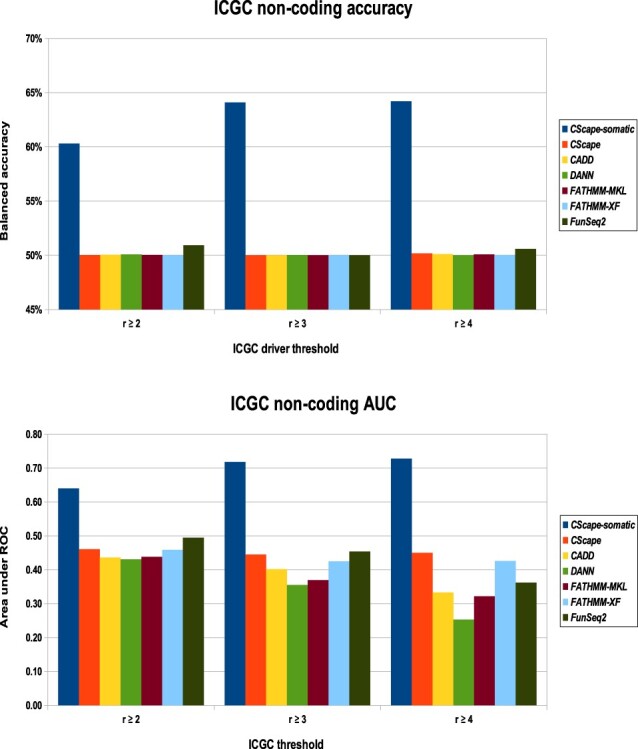
Performance of the best *CScape-somatic* model with the original *CScape*, *CADD* and *FunSeq2* on the ICGC test set for **non-coding** regions (CSS= *CScape-somatic* and CS= *CScape*). Top: *CScape-somatic* yields accuracy from 60.0 up to 64.2% on the ICGC test sets, substantially higher than competitors. The closest competitor changes at each ICGC recurrence level: *FunSeq2* for ICGC r≥2, at 50.9%; *CScape* for ICGC r≥3, at 50.5% and *CADD* for ICGC r≥4, at 51.4%. Bottom: *CScape-somatic* yields AUC scores from 0.64 to 0.73. None of the competitors yield scores better than random chance (0.50), and with the exception of the original *CScape*, perform worse as the driver threshold *r* increases

Generally, we found that *CADD*, which was trained solely on germline or simulated variants, and models such as *CScape*, *FunSeq2*, *DANN*, *FATHMM-MKL* and *FATHMM-XF*, trained on combinations of germline and somatic variants, perform poorly on this test set. *CScape-somatic* yields substantially higher balanced accuracy and AUC scores than competing methods on these data. Interestingly, this model performs better as the recurrence level increases: from 60% at r≥2 up to 64% at r≥4 (Fig. 5). This observation implies there is a substantive difference between low-recurrence and high-recurrence variants, supporting our previously stated assumption that high-recurrence variants are more likely to be driver mutations. The remaining models all perform worse in terms of AUC scores as the ICGC driver threshold *r* increases, the lone exception being the original *CScape* (Fig. 5, bottom).

### 3.5.3 Evaluation on TERT/SDHD/PLEKHS1 examples from non-coding regions

Few oncogenic single-point mutations have been verified in non-coding regions. The most prominent to date are three mutations in the *TERT* promoter region (Horn *et al.*, 2013; Huang *et al.*, 2013; Weinhold *et al.*, 2014). These have been characterized as disruptions to putative *E26 transformation specific* (ETS) family transcription factor binding sites, that include five additional mutations in *SDHD* and *PLEKHS1* (Weinhold *et al.*, 2014). This test set is tiny, and thus inadequate to evaluate classifiers in any comprehensive fashion, but represents the few documented examples of driver mutations in non-coding regions. Hence, we expect a useable classifier to predict a majority of these examples correctly. For both *CScape* methods, we assign negative (-) labels to scores below 0.5 and positive (+) labels for the rest. For CADD scores, we associate negative and positive predictions with negative and positive scores, respectively. For FunSeq2, we label as negative scores below 0.56 and use positive labels for the rest.

The *CScape-somatic* non-coding predictor yield positive predictions for all of these examples, while the original *CScape* predict all but one of the *SDHD* examples (Table 2). *FunSeq2* and *CADD* perform worst in this test, missing both of the *PLEKHS1* examples. However, it is worth repeating that these validated examples represent but a small fraction of cancer drivers in non-coding regions. It is also worth noting that none of these examples appear in the *CScape-somatic* training set, whereas all three of the TERT mutations were part of the original *CScape* training set.

**Table 2.** Tests on verified cancer drivers from non-coding regions show that *CScape-somatic* predicts all variants correctly, while the original *CScape* correctly predicts all but one SDHD variant

Mutation	CSS	CS	FS^a^	CADD
TERT				
5:g1295228G>A	+ (0.56)	+ (0.52)	+ (1.33)	+ (0.34)
5:g1295229G>A	+ (0.51)	+ (0.62)	+ (1.69)	+ (0.66)
5:g1295250G>A	+ (0.51)	+ (0.58)	+ (0.56)	+ (0.31)
SDHD				
11:g111957523C>T	+ (0.52)	+ (0.81)	+ (1.00)	+ (1.64)
11:g111957541C>T	+ (0.68)	+ (0.67)	+ (1.62)	+ (0.82)
11:g111957544C>T	+ (0.87)	− (0.40)	+ (1.00)	+ (0.64)
PLEKHS1				
10:g115511590G>A	+ (0.71)	+ (0.65)	− (0.17)	− (-0.10)
10:g115511593C>T	+ (0.57)	+ (0.71)	− (0.17)	− (-0.06)

*Note*: *FunSeq2* and *CADD* predict the *TERT* and *SDHD* examples correctly, but both misclassify the *PLEKHS1* examples. For each method, we present the predicted label (+ = driver, – = passenger) with the associated score in parentheses. (Classifiers: CSS = *CScape-somatic*, CS = *CScape*, FS = FunSeq2.)

a For FunSeq2, we use a threshold of 0.56 (Rogers *et al.*, 2017a).

### 3.6 X indicates coding

#### 3.6.1 Classifying somatic variants in coding regions

**Fig. 6.**
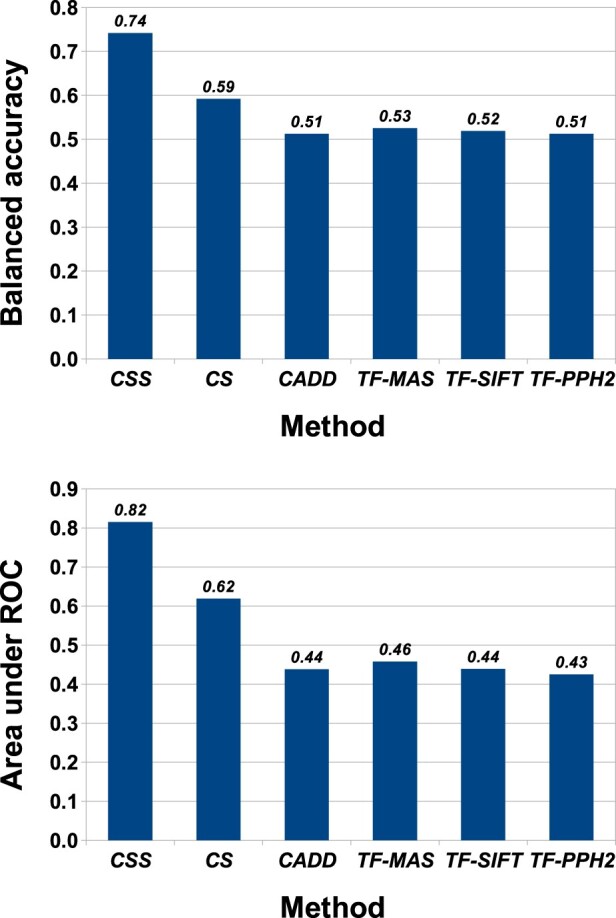
Comparison between *CScape-somatic* performance using LOCO-CV (**coding** regions, **COSMIC** data) with prediction results for *CScape*, *CADD* and *TransFIC* (Gonzalez-Perez *et al.*, 2012) models on the same examples. Top: *CScape-somatic* balanced accuracy in LOCO-CV outperforms other methods on the COSMIC training data, with accuracy over 74%. Of the other methods, only *CScape* yields prediction accuracy better than chance, at 59.2%. The remaining methods fare less well, even the TransFIC methods that were optimized for somatic variants. Bottom: We see the same trend with ROC scores, as *CScape-somatic* yields satisfactory ranking performance of 0.82, whereas only the original *CScape* yields rankings better than chance, at 0.62. (CSS= *CScape-somatic*; CS= *CScape*; TF-MAS= *TransFIC-MutationAssessor*, TF-PPH2= *Transfic-Polyphen2* and TF-SIFT= *TransFIC-SIFT*)

For classifying driver mutations, coding regions have received considerably more attention than non-coding regions. However, few models have been developed expressly to differentiate between somatically acquired cancer drivers and passenger mutations. Hence, we are interested in seeing whether a classifier trained on rare putative passengers and highly recurrent putative drivers in coding regions can discriminate between these two classes, better than existing models. Results on our COSMIC training data, shown in Figure 6, show that most methods struggle to make this distinction. Of the methods tested, only the original *CScape* yields prediction accuracy better than chance, at 56% with an AUC score of 0.62. By contrast, *CScape-somatic* achieves an average balanced accuracy of 74% in LOCO-CV, with an average AUC of 0.82.

**Fig. 7.**
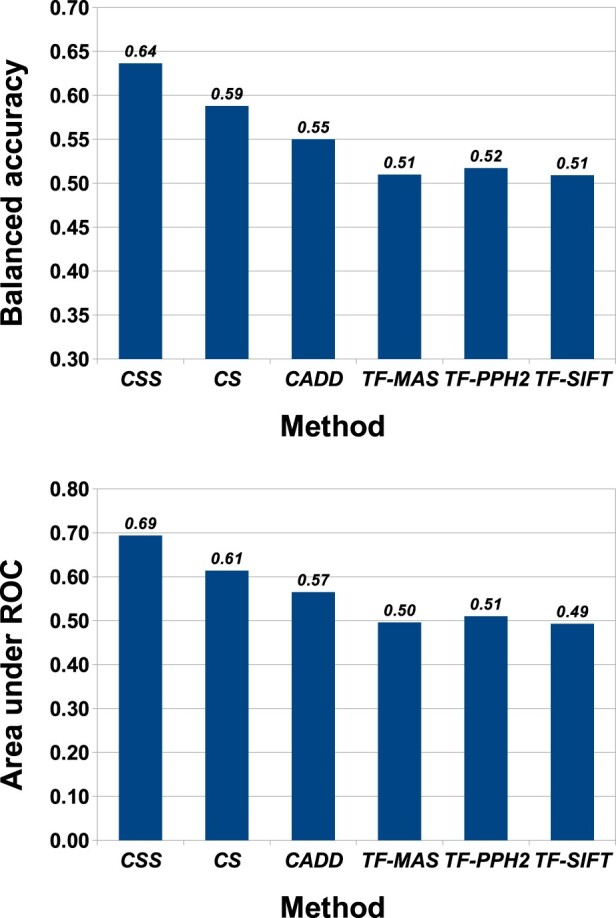
Performance of the best *CScape-somatic* model with the original *CScape*, *CADD* and *TransFIC* models on the **ICGC test set** for **coding** regions. Top: *CScape-somatic* yields substantially higher accuracy, at over 63%, than the original *CScape* at 59%. The remaining methods fare less well, with accuracies ranging from 51 to 56%. Bottom: When consider ranking performance, *CScape-somatic* again outperforms the other methods with an AUC score of 0.69, followed by the original *CScape* at 0.61 and the remaining methods all below 0.60. (CSS= *CScape-somatic*; CS= *CScape*; TF-MAS= *TransFIC-MutationAssessor*, TF-PPH2= *Transfic-Polyphen2* and TF-SIFT= *TransFIC-SIFT.*)

#### 3.6.2 ICGC test data

We see similar performance characteristics on our ICGC test set: the *CScape-somatic* coding classifier yields 64% accuracy and an AUC score of 0.69, whereas the best of the remaining methods, *CScape*, manages 59% accuracy and an AUC of 0.61 (Fig. 7). Taken with the performance on our COSMIC dataset, these results suggest that models trained to discriminate between presumed cancer drivers and generic neutral germline variants may be poor with distinguishing between true drivers and passengers.

We note that the performance of the *CScape-somatic* coding classifier drops considerably between the COSMIC training set and the ICGC test set. By contrast, the original *CScape* performs slightly better on the ICGC test set at 59% accuracy compared with 56% accuracy on the COSMIC dataset. There are two possible reasons for this: either the ICGC test set does not represent cancer drivers and putative passengers as well as the COSMIC dataset, or the *CScape-somatic* coding model may over-fit the COSMIC dataset. After filtering out examples found in our training set, the ICGC test sets are relatively small, with just 1695 drivers and 2921 putative passenger mutations in the set. As a result, we did not have sufficient test data to stratify by recurrence levels with putative drivers defined by recurrence levels as low as two. When we test our coding model on unseen COSMIC data where drivers are identified using recurrence levels of just two or higher, performance indeed drops considerably, to a balanced accuracy of 62.3%, slightly lower than its performance on the ICGC test data using the same recurrence levels. Thus, while we cannot rule out some degree of over-fitting, these results suggest that relatively low recurrence levels in the ICGC data account for some of the observed performance difference.

We have used the COSMIC dataset for model training and the ICGC dataset for test evaluation. Of course, it is also possible to train on ICGC data and test on COSMIC. Though this leads to a slightly lower test performance, we consider and evaluate this alternative in Supplementary Section S5.

Aside from evaluations on test data, we can also test the model for biologically meaningful prediction. There are a number of well-characterized cancer driver mutations stemming from variants in coding regions. For example, the His1047Arg substitution derives from A→G at location 3:178952085 (GRCh37/hg19) in the driver gene *PIK3CA* and has been implicated in various cancers (Janku *et al.*, 2011). Using *CScape-somatic* (http://CScape-somatic.biocompute.org.uk/) this is a high confidence predicted driver (at 0.927). In Supplementary Section S6, we further tested *Cscape-somatic* on a range of other recurring single point driver mutations in coding regions, residing in well-known cancer genes, and characterized as SNV drivers as shown in the Extended Data Figure 1 in the study by Rheinbay *et al.* (2017). Their study uses data from the Pan-Cancer Analysis of Whole Genomes Consortium and uses in excess of 2700 cancer genomes from more than 2500 patients. Subject to the proviso given in Supplementary Section S6, the presented classifier correctly predicts all of these well-characterized drivers from the driver-genes *KRAS*, *PIK3CA*, *TP53*, *NRAS* and *IDH1*.

## 4 Discussion

In this study, we have investigated the feasibility of developing models that can accurately predict the likely influence of different classes of somatic mutations on tumorigenesis. Our hypothesis was twofold. First, there are characteristic differences in many of the features distinguishing rare somatic variants, which are prospectively enriched for neutral passenger variants, and benign germline variants. The latter category is frequently used to train methods for SNV driver status annotation. Second, these features can play an important role in discriminating between rare somatic variants, putatively passengers and highly recurrent somatic variants, restricted to cancer patients, and which are likely to be enriched for drivers. We found evidence to support the first hypothesis within features that measure degree of conservation across the genome, mutation frequency or GC content in the region surrounding each variant. We also present the *CScape-somatic* model to distinguish these two classes of somatic variant in coding and non-coding regions of the genome. Both the coding and non-coding sub-classifiers, optimized separately within their respective domains, rely to some degree on the same features: conservation, mutation frequency and GC content.

To our knowledge, the *CScape-somatic* model is the first to discriminate solely between somatic cancer variants. We compared our new model to our original *CScape* model which was trained to discriminate between somatic driver variants and benign germline variants, and found that while the original model provides weak discrimination between highly recurrent and rare somatic variants, the new model provides substantially higher test accuracy across the entire genome. We also compared this new model to CADD, FunSeq2 and the three TransFIC models: TransFIC-MutationAssessor, TransFIC-SIFT and TransFIC-Polyphen2. Of these latter models, only FunSeq2 has been optimized to predict oncogenic variants. The remaining five methods were all developed to discriminate pathogenic germline variants from benign germline variants. In nearly all cases, we found that models trained on germline variants as the neutral control, were unable to distinguish between highly recurrent putative oncogenic drivers and rare somatic variants, likely to be putative passenger variants. Only models trained on cancer variants, *CScape* and *FunSeq2*, provided weak discrimination on some test data for this type of distinction. 

*Financial Support*: The Integrative Epidemiology Unit is supported by the Medical Research Council (MC_UU_00011/4) and the University of Bristol, and we also acknowledge funding from the Cancer Research UK Integrative Cancer Epidemiology Programme (C18281/A19169).

*Conflict of Interest*: none declared.

## Supplementary Material

btaa242_Supplementary_Data
