# CScape-somatic: distinguishing driver and passenger point mutations in the cancer genome

**DOI:** 10.1093/bioinformatics/btab654

**Published:** 2021-10-25

**Authors:** Mark F Rogers, Tom R Gaunt, Colin Campbell

*Bioinformatics* (2020) doi:10.1093/bioinformatics/btaa242

In the originally published article, a funding acknowledgement was missing. This should read: “*Financial Support*: The Integrative Epidemiology Unit is supported by the Medical Research Council (MC_UU_00011/4) and the University of Bristol, and we also acknowledge funding from the Cancer Research UK Integrative Cancer Epidemiology Programme (C18281/A19169).” instead of: “*Financial Support*: none declared.” These details have been corrected online.

