# Supplementary material for: CScape-somatic: distinguishing driver and passenger point mutations in the cancer genome
Source: Bioinformatics. 2020 Apr 13;36(12):3637–44. doi: 10.1093/bioinformatics/btaa242 (PMC7320610; doi:10.1093/bioinformatics/btaa242)
Supplement: btaa242_Supplementary_Data [file btaa242_supplementary_data.pdf]

# Supplementary material for *CScape-somatic*: distinguishing driver and passenger point mutations in the cancer genome

Mark F. Rogers[1], Tom R. Gaunt[2] and Colin Campbell[1]
[1]Intelligent Systems Laboratory, University of Bristol,
Bristol, BS8 1UB, United Kingdom
[2]MRC Integrative Epidemiology Unit (IEU), University of Bristol,
Bristol, BS8 2BN, United Kingdom

# 1 Method and Data

## 1.1 The Method

The *CScape-somatic* classifier presented in this paper has a similar construction to *CScape* classifier described in Rogers *et al* [9]. Along with the predicted class assignment, the classifier presents an associated confidence measure, or $p$-score, on the range 0 for a negative, to 1 for a positive. Thus 0.8 means an approximate 80% probability of a positive. This $p$-score was calculated via Platt scaling [6]. The *CScape-somatic* predictor is available at *http://cscape-somatic.biocompute.org.uk/*. To construct the classifier we found that gradient boosting [10] gave the best performance on validation data. We used a variety of different data sources (*feature groups*) to train the classifier, which are more fully described in our earlier paper [9], and are also detailed in Supplementary Section 2 below. For example, we used a feature group labelled *conservation*: single nucleotide variants in genomic regions which are highly conserved across species are more likely to be functional in human disease, relative to variants in regions where there has been significant sequence variation across species. To use these feature groups and construct the classifier, we followed a greedy sequential learning approach. That is, we order different feature groups according to prediction accuracy on unseen validation data. We followed a forward selection approach, starting with the best performing feature group. We then select the best combination of this first group with another group, to create a new best grouping, and continue to add subsequent feature groups based on which combinations yielded the highest validation accuracy. We terminate addition of feature groups if the balanced validation accuracy reaches a plateau or starts to decline. This terminates the learning process and then we proceed to evaluation on unseen test data.

## 1.2 The Data

Data were extracted from COSMIC [1], the Catalogue of Somatic Mutations in Cancer, version 84, February 2018 (https://cancer.sanger.ac.uk/cosmic). The training data size is presented in Supplementary Table 1 and is based on the GRCh37/hg19 version of the human genome. As mentioned in the main text, a bias can be introduced if negative examples are located in genomic regions distant from the positive examples (the distinction would becomes more tractable, giving a bias towards higher test accuracy). Consequently, we include only those rare ($r = 1$) somatic variants, labelled as negatives, found within 1,000 (non-coding) or 10,000 (coding) positions from some positive variant.

## 1.3 COSMIC training data

| Chromosome | Coding | | Non-coding | |
|---|---|---|---|---|
| | Pos | Neg | Pos | Neg |
| 1 | 5,258 | 5,183 | 4,077 | 3,429 |
| 2 | 3,735 | 3,566 | 4,261 | 4,020 |
| 3 | 2,973 | 2,686 | 3,458 | 3,102 |
| 4 | 2,090 | 1,902 | 3,381 | 3,338 |
| 5 | 2,800 | 2,641 | 3,445 | 3,279 |
| 6 | 2,566 | 2,441 | 3,001 | 2,501 |
| 7 | 2,747 | 2,641 | 3,405 | 3,393 |
| 8 | 1,983 | 1,830 | 3,154 | 3,318 |
| 9 | 1,898 | 1,724 | 2,199 | 1,972 |
| 10 | 1,936 | 1,683 | 2,412 | 2,296 |
| 11 | 3,120 | 3,355 | 2,408 | 2,161 |
| 12 | 2,742 | 2,359 | 2,240 | 1,925 |
| 13 | 986 | 903 | 1,816 | 1,851 |
| 14 | 1,459 | 1,492 | 1,665 | 1,894 |
| 15 | 1,540 | 1,458 | 1,340 | 1,189 |
| 16 | 1,907 | 1,847 | 1,529 | 1,365 |
| 17 | 2,800 | 2,425 | 1,267 | 822 |
| 18 | 810 | 731 | 1,340 | 1,244 |
| 19 | 3,708 | 4,140 | 1,038 | 897 |
| 20 | 1,382 | 1,286 | 1,087 | 903 |
| 21 | 604 | 484 | 878 | 1,074 |
| 22 | 956 | 900 | 599 | 590 |
| Total | 50,000* | 47,677 | 50,000* | 46,563 |

Supplementary Table 1: The initial set of filtered training data used for these models is largely balanced between positives, recurrent ($r \geq 2$) somatic variants, and the negatives, $r = 1$ somatic variants, for each chromosome and based solely in cancer patient data. We selected negative examples from regions within 1,000 (non-coding) or 10,000 (coding) positions of positives to mitigate location bias. *The COSMIC database contains enough data that we limited our datasets to 50,000 drivers for coding and non-coding regions.

| | Coding ($r \geq 7$) | | Non-coding ($r \geq 8$) | |
|---|---|---|---|---|
| Chromosome | Pos. | Neg. | Pos. | Neg. |
| 1 | 1,487 | 1,483 | 522 | 450 |
| 2 | 813 | 922 | 374 | 435 |
| 3 | 1,182 | 654 | 286 | 254 |
| 4 | 546 | 569 | 214 | 202 |
| 5 | 571 | 911 | 269 | 212 |
| 6 | 877 | 596 | 263 | 195 |
| 7 | 821 | 820 | 404 | 391 |
| 8 | 420 | 582 | 253 | 222 |
| 9 | 583 | 477 | 427 | 305 |
| 10 | 440 | 397 | 272 | 284 |
| 11 | 869 | 928 | 241 | 206 |
| 12 | 747 | 625 | 228 | 145 |
| 13 | 216 | 211 | 123 | 106 |
| 14 | 343 | 449 | 351 | 545 |
| 15 | 416 | 331 | 216 | 200 |
| 16 | 532 | 485 | 226 | 154 |
| 17 | 1,332 | 669 | 195 | 116 |
| 18 | 178 | 224 | 92 | 129 |
| 19 | 1,059 | 1,471 | 126 | 105 |
| 20 | 302 | 285 | 124 | 82 |
| 21 | 143 | 115 | 339 | 260 |
| 22 | 268 | 226 | 174 | 191 |
| Total | 14,145 | 13,430 | 5,719 | 5,189 |

Supplementary Table 2: The final training data used for these models are subsets of the original data, given in Supplementary Table 1. Positive (pos.) examples have a recurrence value $r \geq 7$ for coding regions and $r \geq 8$ for non-coding regions. Negative (neg.) examples have a recurrence level of $r = 1$ in both coding and non-coding regions. Again, these sets are largely balanced between positive variants and negative variants for each chromosome.

## 1.4 ICGC test data

### 1.4.1 Non-coding regions

| Recurrence threshold | Positive | Negative | Total |
|---|---|---|---|
| $r \geq 2$ | 15,023 | 36,187 | 51,210 |
| $r \geq 3$ | 1,548 | 3,582 | 5,130 |
| $r \geq 4$ | 481 | 1,146 | 1,627 |

Supplementary Table 3: ICGC test data for non-coding regions yielded over 50,000 test examples distinct from our COSMIC training data. Patient data annotations allowed us to identify variants that recur up to three times within the database.

The ICGC database [16] provided enough non-coding examples along with patient identifiers that allowed us to assign recurrence values to each variant. Thus we used the same approach with these data as with our COSMIC training set: we labeled variants appearing just once ($r = 1$) as putative passengers (negative) and those with $r > 1$ as putative drivers (positive). We had a sufficient number of positive examples that we were able to create three distinct data sets for drivers with $r \geq 2$, $r \geq 3$, or $r \geq 4$. After removing all variants common between ICGC and the COSMIC data sets, we were left with the data sets shown in Supplementary Table 3.

### 1.4.2 Coding regions

After removing variants common to both the ICGC and COSMIC data sets, we were left with just 4,427 coding examples, including 1,695 positive and 2,732 negative examples. Few of these remaining examples had recurrence levels greater than $r = 2$, so we were unable to investigate performance at more than one recurrence level.

# 2 Features

We used eight distinct feature groups for our two classifiers, five of which are common to both. These are described briefly below.

- **Spectrum kernel**
  To capture the disruption that occurs in the sequence surrounding a mutation, we use *spectrum* kernels [5] that compare $k$-mer composition in windows of size $w$ across a mutation. For single-point mutations the disruption is confined to a relatively small region, so for both coding and non-coding models a window size $w = 3$ and maximum k-mer size 2 yields the best discrimination.

- **Conservation and Evolutionary**
  We use several conservation based measures, such as PhyloP [7], PhastCons [13] and FATHMM [12] scores. These are derived from multiple sequence alignments of 46 or 100 vertebrate genomes to the human genome [2]. In addition, we used dN/dS ratios between human and 65 different species (one-to-one orthologues).

- **GC content**
  Similar to the spectrum kernel, these features represent the percentage GC content for five distinct window sizes of 2, 4, 8, 16 and 32 bases flanking a position. This yields a 10-element vector of percent-GC values: five using the reference allele and five using the variant.

- **Distance from gene features**
  To assess a variant's potential impact on nearby gene products, we measure the distance from each variant to gene features annotated in the ENSEMBL gene models: *start codon*, *stop codon*, *gene*, *UTR*, *CDS* and *exon*. This approach is simple, yet provides an opportunity for models to learn relationships between variants and important gene elements.

- **Mutation tolerance**
  The potential impact of a cancer variant may depend on the tolerance for mutations within its region [4]. We thus provide our classifiers with a count of the number of annotated variants within windows of 100 to 1 million bases flanking a variant, not including the variant itself.

- **Functional elements**
  This feature group encapsulates chromatin state segmentation from the ENCODE "BroadHMM" dataset encompassing nine human cell types. Genome segments that predict functional elements were generated from a combination of computationally integrated ChIP-seq data and input from a Hidden Markov Model (HMM).

- **Mapability**
  This feature group is derived from the ENCODE "Mapability" dataset that incorporates three different measurements and different window sizes to measure sequence uniqueness within a reference genome. A high score indicates an area where sequence is unique.

- **Variant effects**
  For coding regions we derive features from the ENSEMBL Variant Effect Predictor (VEP). The VEP provides characteristics for specific genomic locations that we can exploit to predict the

likely impact of a SNV. Our features represent just two forms of data from this database: (1) consequence types, such as *missense variant*, *nonsense variant* or *TF binding site variant* and (2) the amino acid composition of the reference and allele residues.

For more thorough descriptions of these feature groups, see [9].

To identify the most effective recurrence levels for identifying positive training examples, we used complete forward-selection training procedures for coding and non-coding models, using values from $r \geq 2$ to $r \geq 8$ (coding regions) or $r \geq 9$ (non-coding) for positive examples. As shown in Figure 1(top) of the main paper, we found that balanced accuracy increased substantially for recurrence levels up to $r \geq 6$ for coding regions, reaching a nominal peak with $r \geq 7$. For non-coding regions, performance increased steadily from $r \geq 2$ up to $r \geq 8$ before declining again at higher recurrence levels. For our models we thus selected $r \geq 7$ as positive examples for coding regions, and $r \geq 8$ for non-coding regions.

## 2.1 Final feature sets

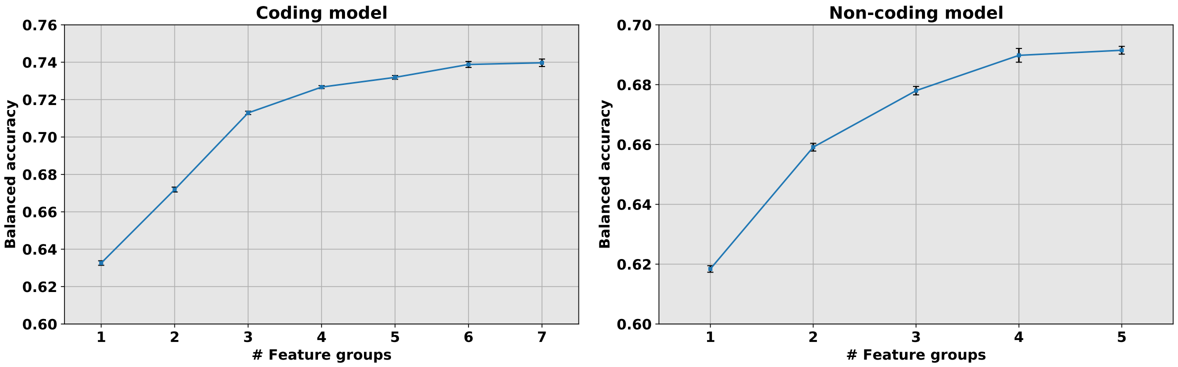


Supplementary Figure 1: Progress of the forward selection process shows that balanced accuracy increases steadily for both coding (left) and non-coding (right) models. **Left:** coding model balanced accuracy increases from 63.3% to 74.0% as we add the first six feature groups, but plateaus at that point. **Right:** non-coding model balanced accuracy increases from 61.8% to 69.0% as we add the first four feature groups, but plateaus at the fifth group at 69.2%. Error bars represent 95% confidence intervals over 10 LOCO-CV runs.

Supplementary Figure 1 shows the progress of our forward selection procedure as individual feature groups are added to each of our models. Each model was tested using LOCO-CV, using balanced training sets of $4,000$ positive and $4,000$ negative from 21 chromosomes and testing on the remaining chromosome (test set composition shown in Supplementary Table 1). For both models, balanced accuracy increased steadily as we added feature groups. Coding model performance peaked at six feature groups with balanced accuracy just over 74% (Supplementary Figure 1, right). Forward selection identified feature groups for the coding model in the following order: 1=*Spectrum kernel*, 2=*Mutation tolerance*, 3=*46-way conservation*, 4=*Chromatin state*, 5=*GC content*, 6=*Mapability* and 7=*VEP*. Since there was no statistically significant difference between the last two models, only the first six groups were included in the final model.

Non-coding performance also increased steadily with a nominal peak at five feature groups with balanced accuracy at just over 69% (Supplementary Figure 1, left). Forward selection identified feature groups for the non-coding model as follows: 1=*100-way conservation*, 2=*Mutation tolerance*, 3=*GC content*, 4=*Mapability* and 5=*Distance from gene features*. Coding models used $r \geq 7$ as positive examples, while non-coding models used $r \geq 8$.

# 3 Comparison of benign germline variants and rare ($r = 1$) somatic variants which are likely enriched as passengers

A key aspect of our motivation for exploring models based solely on somatic variants was our hypothesis that there would be characteristic differences between neutral germline variants and putative somatic passenger variants (occurrence of $r = 1$). We thus evaluated feature values for assumed neutral variants from the 1000 Genomes database [14] and compared those with the same feature values for putative somatic passenger variants from the COSMIC database. We found a considerable number of features that exhibited different characteristics between these two populations.

## 3.1 Non-coding regions

In non-coding regions, at least three feature groups from the final models exhibit noticeable differences between putative germline neutral variants and somatic neutral variants (Supplementary Figure 2). The *Mutation tolerance* feature group provides simple counts of the number of somatic variants (both drivers and passengers) within the regions flanking a position. For smaller windows (100 to 1,000 positions) somatic variants are extremely rare around neutral germline variants, but appear more frequently near putative somatic passenger variants. This trend continues as we increase the window size up to 100,000 positions, although the distinctions become less obvious; in fact the final models include only the features for smaller windows. Within the *Conservation* group we include features such as FATHMM, PhastCons and PhyloP scores as well as constituents of the FATHMM score: HMMER state transition probabilities for wild-type (pW) and mutant (pM) alleles, and the difference between them. Finally, *GC content* reveals subtle differences in the frequencies, particularly in the regions surrounding wild-type alleles, with neutral germline variants appearing more frequently in low-GC content regions than putative passenger somatic variants ($r = 1$).

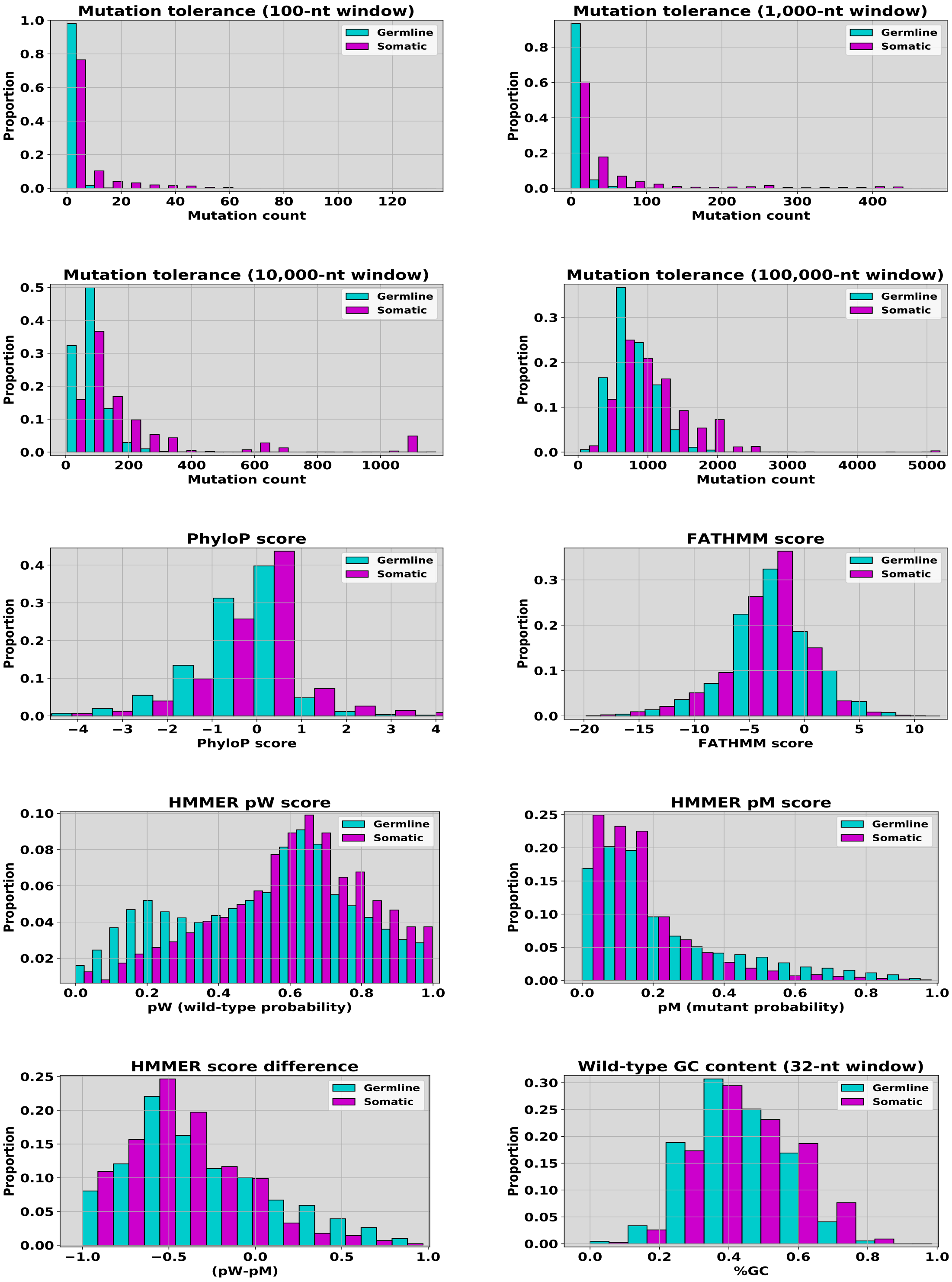


Supplementary Figure 2: Differences in feature distributions for germline and rare ($r = 1$) somatic examples in **non-coding** regions supports our hypothesis that there may be characteristic differences between these two kinds of "benign" variants. All differences in these distributions are statistically significant at $p < 10^{-18}$ or below (by a Mann-Whitney test).

### 3.2 Coding regions

In coding regions, we again see differences in the *Conservation* and *GC content* groups. Within the *Conservation* group, the three precomputed conservation scores PhastCons, PhyloP and FATHMM all exhibit noticeable differences between germline and somatic variants. Similarly, there are differences in the HMMER state transition probabilities pW and pM as well. GC content scores exhibit differences in the regions immediately surrounding variants. In contrast to non-coding variants, it is the somatic variants that appear more frequently in regions with low GC content.

## 3.3 Coding regions

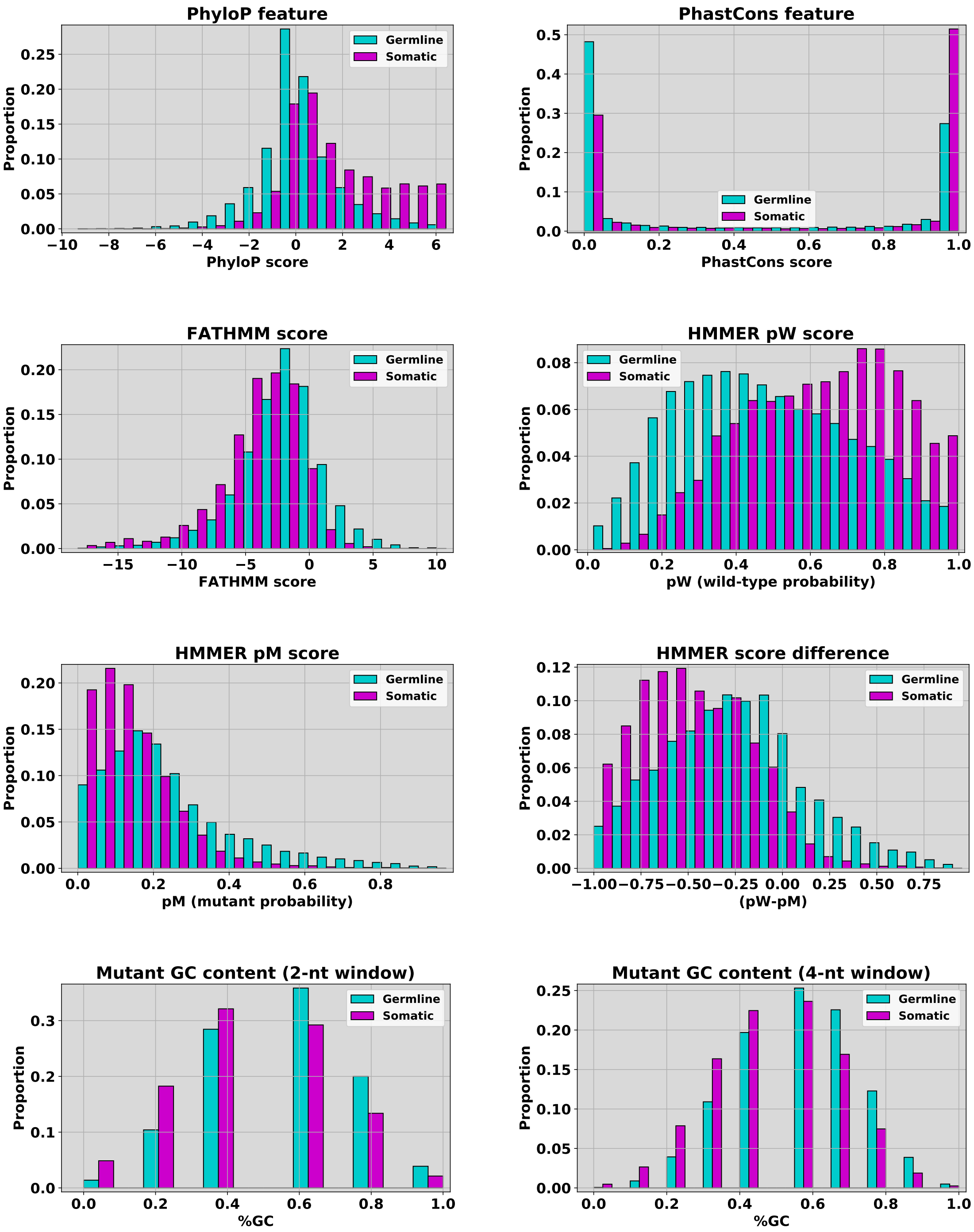


Supplementary Figure 3: Differences in feature distributions for germline and somatic neutral examples in **coding** regions again appears to support our hypothesis that there are characteristic differences between neutral germline and neutral somatic variants. All differences in these distributions are statistically significant at $p < 10^{-40}$ or below (Mann-Whitney).

### 3.4 Classifying non-drivers between germline and somatic variants

To complete the picture of the potential differences between rare ($r = 1$) somatic variants, which are likely putative passengers, and benign germline variants, we constructed classifiers using the same kinds of features used in the respective *CScape-somatic* models for coding and non-coding regions. These feature groups include all of the features shown in the preceding two sections. In coding regions, the model achieved 74% balanced accuracy and 0.82 AUC (Figure 4, left), while for non-coding regions the model had balanced accuracy of 62% and 0.67 AUC (Figure 4, right). It is worth reiterating that these models used the same feature groups as the *CScape-somatic* models: stronger performance might be achieved using our forward selection procedure on each of these. However, our goal was to evaluate characteristic differences between these two sets of neutral variants in the context of our *CScape-somatic* classifiers.

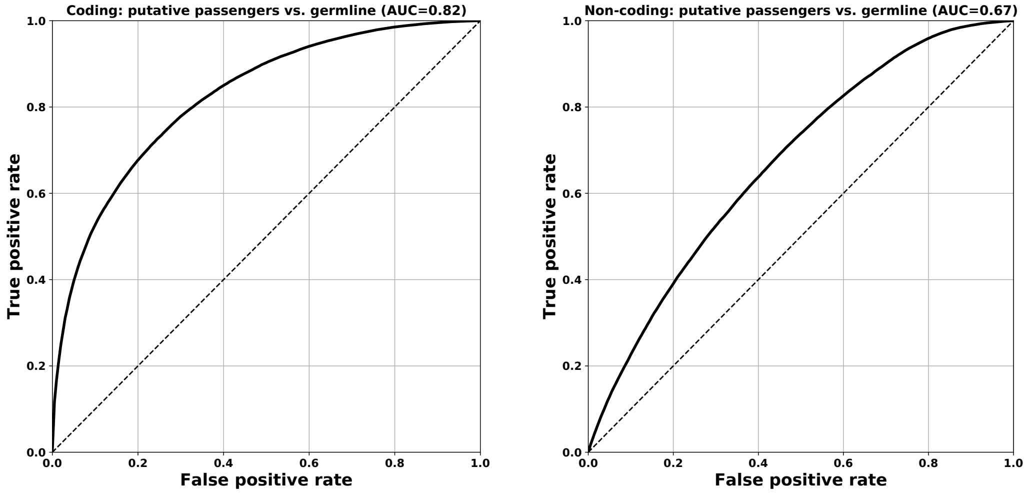


Supplementary Figure 4: Classifiers trained to discriminate between putative somatic passenger variants (COSMIC variants with $r = 1$) and benign germline variants from 1000 Genomes exhibit strong discriminatory performance, with 74% balanced accuracy and 0.82 AUC in coding regions (left), and balanced accuracy of 62% and 0.67 AUC in non-coding regions (right).

# 4 Cautious Classification

As in our previous studies [11, 9], we establish thresholds for each classifier that yield the highest possible accuracy by restricting our attention only to high-confidence predictions. By varying a threshold $\tau$ through the range of possible values from the default threshold at 0.5 up to the maximum 1.0, a model's accuracy tends to increase monotonically until it reaches a peak that may be considerably higher than its accuracy at the default threshold. The non-coding classifier reaches its peak at $\tau = 0.84$, predicting 19% of examples at a balanced accuracy of 84.2% (Figure 5, top). The coding classifier achieves peak balanced accuracy of 92% at a threshold of $\tau = 0.91$, yielding predictions for 10% of examples (Figure 5, bottom).

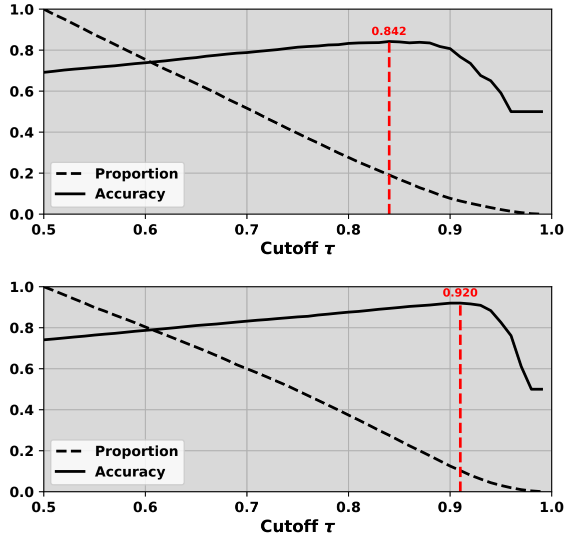


Supplementary Figure 5: **Cautious classification** that restricts predictions only to high-confidence scores can yield substantially higher accuracy than the default prediction threshold of $\tau = 0.5$. **Top:** the non-coding classifier yields up to 84.2% accuracy at a threshold of $\tau = 0.84$. **Bottom:** the coding classifier yields 92.0% accuracy at a threshold of $\tau = 0.91$.

# 5 An alternative model with ICGC as training data and COSMIC as the test data

Our procedure for training models on COSMIC examples and then testing on a separate data set such as ICGC relies on the suitability of COSMIC recurrence levels for predicting high-recurrence variants, that we interpret as drivers. However other data sets such as ICGC provide enough information to infer recurrence levels, and hence could be used as the basis for a alternative model. As noted in the main paper, we found a sufficient number of examples in the ICGC database to evaluate the distinction between recurrence levels $r = 1$ and $r \geq 2$ in coding regions, and between $r = 1$ and $r \geq 2$, $r \geq 3$ or $r \geq 4$ in non-coding regions. Hence we can apply the same model construction but with ICGC as training data so as to evaluate the predictive power of models based on ICGC data instead.

Models trained on ICGC data did not perform as well in LOCO-CV, nor in testing on the COSMIC examples (Table 4). In coding regions, balanced accuracy in LOCO-CV declined by up to six percentage points, with ICGC-trained models yielding 68.2% balanced accuracy compared with 74.2% accuracy for COSMIC-trained models. In non-coding regions, the differences were less obvious, with balanced accuracy in LOCO-CV ranging from 62.4% to 68.5% for ICGC-trained models, compared with 69.2% for the final COSMIC-trained non-coding model. We also found a substantial decline in performance when testing the ICGC-trained models against COSMIC examples. This may be due to some important differences in the feature groups used in the ICGC-trained models.

A prominent difference in these feature groups is the reliance on ICGC data, rather than COSMIC data, to obtain mutation frequencies (Table 5). This was necessary to mitigate potential bias in ICGC-trained models by preventing them from "learning" anything about the COSMIC data prior to testing on the COSMIC examples. However, the ICGC database contains 3.5 million distinct point mutations compared to nearly 10.8 million in the COSMIC dataset. Hence there is a slightly higher capacity for models to learn mutation frequencies specific to ICGC that may not translate to other data sets; in other words, these models may tend to overfit to ICGC mutation frequencies. The final selection of feature groups for ICGC models given in Table 5 shows that, as with the COSMIC models,

| Test set | ICGC-based models | | | |
|---|---|---|---|---|
| | | Non-coding | | |
| | Coding | $r \geq 2$ | $r \geq 3$ | $r \geq 4$ |
| LOCO-CV | 0.682 | 0.624 | 0.685 | 0.682 |
| COSMIC ($r \geq 2$) | 0.577 | 0.538 | 0.526 | 0.522 |
| COSMIC ($r \geq 3$) | 0.575 | 0.563 | 0.555 | 0.535 |
| COSMIC ($r \geq 4$) | 0.571 | 0.571 | 0.571 | 0.547 |
| COSMIC ($r \geq 5$) | 0.581 | 0.588 | 0.592 | 0.559 |
| COSMIC ($r \geq 6$) | 0.590 | 0.604 | 0.604 | 0.573 |
| COSMIC ($r \geq 7$) | **0.592** | 0.594 | 0.596 | 0.566 |
| COSMIC ($r \geq 8$) | 0.586 | **0.591** | **0.592** | **0.570** |
| COSMIC ($r \geq 9$) | 0.584 | 0.581 | 0.583 | 0.567 |

Supplementary Table 4: Balanced accuracy for models trained on ICGC examples in LOCO-CV (top row) and tested on COSMIC examples distinct from any ICGC training data (remaining rows). For coding regions, performance falls well short of the final models trained on COSMIC data, where balanced accuracy in LOCO-CV was up to 74.2%, compared with 68.2% for models trained on ICGC examples. Performance in non-coding regions is more comparable, with the best ICGC-based models reaching balanced accuracy of 68.2% and 68.5% for $r \geq 3$ and $r \geq 4$, respectively, compared with 69.2% accuracy for the COSMIC-based model. We see a similar pattern when we test these models on distinct COSMIC examples: for coding regions, balanced accuracy peaks at 59.2% tested on COSMIC $r \geq 7$ examples, while the COSMIC-trained model yields 63.6% accuracy tested on ICGC examples. In non-coding regions, test accuracy on COSMIC examples ranges from 52.2% to 60.4%, while COSMIC models predict ICGC examples with 60.3% to 64.2% accuracy in these regions.

| Model | Feature groups |
|---|---|
| Coding | *ICGC mutation frequency, Spectrum, Distance* |
| Non-coding ($r \geq 2$) | *ICGC mutation frequency, Spectrum, Conservation, GC content, Sequence uniqueness* |
| Non-coding ($r \geq 3$) | *ICGC mutation frequency, Spectrum, Conservation, GC content, Sequence uniqueness* |
| Non-coding ($r \geq 4$) | *ICGC mutation frequency, Spectrum, Conservation* |

Supplementary Table 5: Above are shown the feature groups selected for the best ICGC-trained models based on the same forward selection procedure used with the COSMIC-trained models. The most prominent feature group is mutation frequency based on the ICGC database, followed by the spectrum kernel. For variants in coding regions, distance from gene features such as start/stop codons and transcription start sites is also informative; in non-coding regions, conservation scores, GC content and sequence uniqueness are more predictive of high-recurrence variants.

conservation scores, GC content and sequence uniqueness also play an important role.

# 6 Evaluation of *CScape-somatic* on characterised common recurrent driver mutations

In this section we report performance on some of the top top recurring single point driver mutations in coding regions proposed by Rheinbay *et al* (Extended Data Figure 1 in [8]). The predictions are for characterised drivers in well-known cancer genes which are typically also marked by a high recurrence within tumours. We consider all of their recurring mutations labeled by *CScape-somatic* as lying within coding regions and excluding the allosomes (X and Y). Entries in Supplementary Table 6 are the confidences for assignment of the single nucleotide variant to driver-status.

| Gene | Chr. | Position | Ref. | Mut. | Confidences |
|---|---|---|---|---|---|
| *KRAS* | 12 | 25398284 | C | {A,G,T} | {0.904,0.891,0.903} |
| *KRAS* | 12 | 25398285 | C | {A,G,T} | {0.861,0.900,0.877} |
| *PIK3CA* | 3 | 178952085 | A | {C,G,T} | {0.924,0.928,0.896} |
| *PIK3CA* | 3 | 178936091 | G | {A,C,T} | {0.841,0.871,0.841} |
| *TP53* | 17 | 7577120 | C | {A,G,T} | {0.972,0.976,0.970} |
| *TP53* | 17 | 7577538 | C | {A,G,T} | {0.944,0.970,0.957} |
| *TP53* | 17 | 7577094 | G | {A,C,T} | {0.951,0.954,0.942} |
| *TP53* | 17 | 7578212 | G | {A,C,T} | {0.995,0.991,0.991} |
| *TP53* | 17 | 7578190 | T | {A,C,G} | {0.954,0.956,0.955} |
| *TP53* | 17 | 7577539 | G | {A,C,T} | {0.950,0.965,0.947} |
| *TP53* | 17 | 7577121 | G | {A,C,T} | {0.964,0.972,0.966} |
| *NRAS* | 1 | 115256530 | G | {A,C,T} | {0.604,0.675,0.592} |
| *IDH1* | 2 | 209113112 | C | {A,G,T} | {0.801,0.846,0.800} |

Supplementary Table 6: The top recurring single point driver mutations in coding regions proposed by Rheinbay *et al* (Extended Data Figure 1 in [8]). Their study uses data from the Pan-Cancer Analysis of Whole Genomes Consortium and uses in excess of 2,700 cancer genomes from more than 2,500 patients. This table only gives single nucleotide variants located on autosomes and labeled by our classifier as residing in coding regions. The table presents the gene, chromosome (Chr.), position and reference nucleotide (Ref.) based on the GRCh37 reference genome. The three prospective variants are presented (Mut.) with the confidence driver-status given in the next column, in the same relative order, and derived from our predictor *CScape-somatic* (*http://cscape-somatic.biocompute.org.uk*).

Though *CScape-somatic* gives the correct prediction label in all instances, this Table should be regarded *as a validity check*. These SNVs are well characterised examples of drivers and have a high recurrence rate across a large sample of tumours. The Pan-Cancer Analysis of Whole Genomes (PCAWG) Consortium dataset derives from a combination of the International Cancer Genome Consortium (ICGC) [16] and The Cancer Genome Atlas (TCGA) [15] datasets. This is separate dataset from that used to train *CScape-somatic*, i.e. the COSMIC database [3]. However, highly recurrent SNVs in the PCAWG data are likely to be highly recurrent in the COSMIC database. If a classifier is trained to zero training error on its training set, then the re-presentation of a training example will be classified correctly. In short, these well characterised examples from the PCAWG dataset may effectively present as training examples and correct classification could be expected. Hence, the presented Supplementary Table 6 is to demonstrate that the method correctly classifies well characterised examples of SNV-drivers. In the main text, though, performance is evaluated via *test* evaluation i.e. on data which is unseen to the classifier. This is implemented by, for example, LOCO-CV in which the held-out chromosome equates to unseen data for classifier evaluation.

## References


[1] http://cancer.sanger.ac.uk/cosmic/help/gene/analysis.

[2] Mathieu Blanchette, W James Kent, Cathy Riemer, Laura Elnitski, Arian FA Smit, Krishna M Roskin, Robert Baertsch, Kate Rosenbloom, Hiram Clawson, Eric D Green, et al. Aligning multiple genomic sequences with the threaded blockset aligner. *Genome Research*, 14(4):708–715, 2004.

[3] Simon A Forbes et al. Cosmic: mining complete cancer genomes in the catalogue of somatic mutations in cancer. *Nucleic Acids Research*, 39(suppl_1):D945–D950, 2010.

[4] Abel Gonzalez-Perez, Jordi Deu-Pons, and Nuria Lopez-Bigas. Improving the prediction of the functional impact of cancer mutations by baseline tolerance transformation. *Genome Medicine*, 4(11):1, 2012.

[5] C. S. Leslie, E. Eskin, and W. S. Noble. The spectrum kernel: A string kernel for SVM protein classification. In *Pacific Symposium on Biocomputing*, volume 7, pp. 566–575. World Scientific, 2002.

[6] J. Platt. Probabilistic outputs for support vector machines and comparison to regularised likelihood methods. In *Advances in large margin classifiers*, pp. 61–74. MIT Press, 1999.

[7] K. S. Pollard, M.J. Hubisz, K.R. Rosenbloom, and A. Siepel. Detection of non-neutral substitution rates on mammalian phylogenies. *Genome Research*, 20:110–121, 2010.

[8] E. *et al.* Rheinbay. Discovery and characterization of coding and non-coding driver mutations in more than 2,500 whole cancer genomes. *bioRxiv working paper*, 2017.

[9] Mark F Rogers, Hashem A Shihab, Tom R Gaunt, and Colin Campbell. CScape: a tool for predicting oncogenic single-point mutations in the cancer genome. *Scientific Reports*, 7(1):11597, 2017.

[10] Robert E Schapire. The boosting approach to machine learning: An overview. In *Nonlinear estimation and classification*, pp. 149–171. Springer, 2003.

[11] H. Shihab, M. Rogers, J. Gough, M. Mort, D. Cooper, I. Day, T. Gaunt, and C. Campbell. An integrative approach to predicting the functional effects of non-coding and coding sequence variation. *Bioinformatics*, 31:1536–1543, 2015.

[12] Shihab, H.A. *et al.* Predicting the functional, molecular and phenotypuc consequences of amino acid substitutions using hidden markov models. *Hum. Mutat.*, 34:57–65, 2013.

[13] A. Siepel, G. Bejerano, J.S. Pedersen, A.S. Hinrichs, M. Hou, K. Rosenbloom, H. Clawson, J. Spieth, L.W. Hillier, S. Richards, G.M. Weinstock, R.K. Wilson, R.A. Gibbs, W.J. Kent, W. Miller, and D. Haussler. Evolutionarily conserved elements in vertebrate, insect, worm, and yeast genomes. *Genome Research*, 15:1034–1050, 2005.

[14] The 1000 Genomes Project Consortium. An integrated map of genetic variation from 1,092 human genomes. *Nature*, 491:56–65, 2012.

[15] John N Weinstein *et al.* *Nature Genetics*, 45(10):1113–1120, 2013.

[16] Junjun Zhang, Joachim Baran, Anthony Cros, Jonathan M Guberman, Syed Haider, Jack Hsu, Yong Liang, Elena Rivkin, Jianxin Wang, Brett Whitty, et al. International cancer genome consortium data portal – a one-stop shop for cancer genomics data. *Database*, 2011, 2011.
